# Factors Associated With Patient Ratings of Timeliness of Primary Care Appointments

**DOI:** 10.1177/2374373520968979

**Published:** 2020-10-27

**Authors:** Michael Mayo-Smith, Laurel E. Radwin, Hassen Abdulkerim, David C. Mohr

**Affiliations:** 1 Department of Medicine, Geisel School of Medicine at Dartmouth, Hanover, NH, USA; 2 Center for Primary Care, Harvard Medical School, Boston, MA, USA; 3 Center for Healthcare Organization and Implementation Research (CHOIR), VA Boston Healthcare System, Boston, MA, USA; 4 Department of Health Law, Policy & Management, Boston University School of Public Health, Boston, MA, USA

**Keywords:** primary care, appointments, timeliness, access, patient experience, veterans

## Abstract

As access is the lowest rated dimension in surveys of outpatient experience, we sought to identify patient, practice, and provider factors associated with positive ratings of timeliness of primary care appointments. A cross-sectional study with multivariable, multilevel logistic regression was performed using survey responses from 236 695 individuals receiving care in the Veterans Health Administration (VA). Top box ratings (response of “always”) for whether the patient reported receiving an appointment as soon as they needed in primary care for routine care and for care needed right away were the main outcomes. Independent variables capturing patient, practice, and provider factors were obtained from survey responses and VA databases. Degree of continuity with primary care provider and duration of relationship were strongly associated with higher ratings. Shorter primary care appointment wait times for both new and returning patients were associated with higher ratings. Independent wait times for mental health and specialty appointments had no effect. Older age, better self-reported physical and mental health, lower disease complexity, and rural residence were patient factors associated with higher ratings while gender, race, ethnicity, and education had little effect. Measures of continuity with primary care provider as well as appointment wait times have strong association with positive patient ratings of appointment timeliness. Patients treated in Veterans Affairs clinics may value continuity with their primary care provider over longer times. Initiatives to improve access could focus on improving continuity and ensuring efforts to improve access do not impact continuity.

## Introduction

The National Academy of Medicine’s publication *Crossing the Quality Chasm: A New Health System for the 21st Century* identified timeliness as a key domain of health care quality (1). In primary care, a key element of timeliness is the ability to get appointments as soon as needed. Outpatient surveys routinely include questions on this issue and the results are widely used, including public reporting on Center for Medicaid Services Health Plan Compare and Physician Compare websites (2,3), in the Health Plan star rating system(4), in quality score affecting payments to Accountable Care Organizations (5), and physician pay for performance (6). However, scores from these surveys on the domain of access, which include the questions on appointment timeliness, have been the lowest rated area for outpatient care, with primary care scoring even lower than specialists (7). Based on our review published reports, there has been little or no improvement over the last 4 years (8).

Despite its importance, research on what influences patient ratings of appointment timeliness is limited. Not surprisingly, investigations have shown ratings are impacted by wait times for appointments. In 2 studies of elective orthopedic surgery, patients were more likely to be dissatisfied as the waiting time increased (9,10). Other investigators found shorter appointment wait times in the Veterans Health Administration (VA) were positively associated with ratings of appointment timeliness (11,12).

We hypothesized that patient factors, provider characteristics, and practice variables impact patient ratings of appointment timeliness. Understanding these factors could identify additional avenues for improvement efforts and designing a delivery system better attuned to the preferences and values of patients. To test this relation, we developed multivariable models examining the association of specific factors with ratings of appointment timeliness.

## Methods

The study was a cross-sectional analysis of national survey data on patient experiences in VA primary care, approved by the Boston VA Healthcare System Institutional Review Board

### Participants

Patients were surveyed between September 2015 to October 2016. Patient data were obtained through an internal Data Use Agreement with the VA Office of Reporting, Analytics, Improvement & Deployment. We obtained individual-level patient survey responses based on approval of an internal Data Use Request. Patients were randomly selected from each practice location based on number of providers per site. Patients from all VA medical centers distributed in more than 140 locations and more than 1000 community-based outpatient clinics throughout United States and territories were included. Patients were eligible to receive a survey if they were Veterans, 18 years or older, and had a primary care visit with an assigned provider. No exclusions were made on the basis of medical conditions, insurance, or language. Patients were excluded if they had only telehealth visits or were receiving hospice or palliative care or residing in a noncommunity setting, responded to another VA questionnaire in the previous 12 months, or requested not to be surveyed.

### Measures

#### Patient rating of appointment timeliness

The VA Survey of Healthcare Experiences of Patients (SHEP) - Patient-Centered Medical Home (PCMH) is modeled on the Consumer Assessment of Healthcare Providers and Systems Clinician and Group Survey which assesses patients’ experiences with health care providers and staff in doctor’s offices (13). Results are summarized using 6 composite measures. The access composite consists of 6 questions with 2 asking about timeliness of appointments, the focus of this study. The first question reads: “In the last 6 months, when you contacted this provider’s office to get an appointment for *care you needed right away*, how often did you get an appointment as soon as you needed?” The second reads: “In the last 6 months, when you made an appointment for a *check-up or routine care* with this provider, how often did you get an appointment as soon as you needed?” There were 4 possible responses: never, sometimes, usually, and always. “Top box” responses (“always”) were assigned a value of “1,” and others a value of “0,” consistent with how data are reported nationally.

#### Patient factors

Using administrative data, we modeled patient *age and sex*. We first created Elixhauser comorbidities (8) and coded patients as having 0, 1, 2, or 3 or more comorbid conditions. Using self-reported responses on the survey, we modeled *race, ethnicity, education, language other than English spoken at home*, and a global rating of *general health and mental health*. *Rurality* was measured as the percentage of primary care patients at a given practice location who lived in rural or highly rural areas and was obtained from administrative databases.

#### Provider factors

Respondents indicated if the person named on the survey as their most recent provider seen was their *usual provider*. Respondents also indicated the number of *provider visits* to the same provider they had in the prior 6 months. *Provider relationship length* was measured based on the number of years the respondent reported seeing the provider. *Provider continuity* was measured as the percentage of primary care encounters with a patient’s assigned provider, using the average for practice location from administrative sources.

#### Practice factors

Measures were modeled using performance for the given practice location. Postdischarge contact reflected percentage of patients contacted within 2 business days by primary care team following hospital discharge. *Emergency room utilization* reflected the number of emergency room encounters divided by number of primary care patients. *Nontraditional encounters* reflected telephone, group, or secure messaging encounters per total team assignments. *Support staff ratio* was measured as primary care support staff per primary care provider full-time equivalent employee. *Panel size ratio* was measured as observed to expected number of patients per physician based on VA policy (14). Patients on a team with values of 1 indicated a balanced panel and values >1 indicated more patients than expected. Given that patients could have multiple appointments outside of primary care that could influence access ratings, we modeled wait times by practice location. Based on research examining different measures of wait time in relation to patient ratings of appointment timeliness (12), we selected measures of wait time for new and established patients. For returning patients, the *prospective, desired date* was computed as the difference in days between scheduled appointment date and date desired by the patient. For new patients, *retrospective, create date* was computed as the difference between completed appointment date, and the date the appointment was created in the scheduling system. Measures were obtained for primary care, mental health, and specialty care. We modeled *same day appointments* for primary care and the *third next available appointment*, *defined as days between when a request for an appointment was made and the third available appointment* (15). *Community-based outpatient clinic* was coded to reflect whether the patient was seen at a medical center or free-standing outpatient clinic. *Facility complexity* was modeled to account for factors such as patient volume, complexity, teaching, and research activity (16). We also accounted for hospital network to reflect regional, administrative, and policy differences in medical practice style (17).

### Analysis

We first examined descriptive statistics to assure the items met the assumptions of the analysis and examined correlations for potential multicollinearity concerns. Multivariable logistic regression modeling was conducted where patients were clustered by location to account for the influence of higher-level variables, such as clinic wait times on individual survey responses. We group-mean centered practice location level variables. We reported adjusted odds ratios (ORs) and 95% CIs. As a sensitivity analysis, we created quartiles of practice location measures to examine if nonlinear relations exist. Analysis was conducted using SAS software 9.4.

## Results

The national response rate to the primary care outpatient survey was 40.5% (n = 320,620 respondents). A total of 224 585 respondents matched across data sets and answered at least one of the 2 access items, with 203 884 responses for routine care and 98 484 for care needed right away. Participants needed to first indicate on a prior question that they requested an urgent care appointment before answering, leading to a lower number of respondents. Comparing respondents and nonrespondents, we found groups were relatively similar on demographic characteristics with virtually no difference at the clinic level. Patients with lower self-rated health and higher comorbidity scores were slightly more likely to answer the care needed right away question.

Descriptive statistics of factors from survey data are presented in Table 1. Table 2 provides descriptive statistics of measures from VA databases, reported at the practice level. Correlations were <.50 among clinic variables, and variance inflation factor values tested in linear regression models were <2.2, indicating limited collinearity concerns.

**Table 1.** Distribution of Responses From Survey.^a^

Patient factors	Frequency	Percent	Provider factors	Frequency	Percent
Age			Provider visits within past 6 months		
Less than 45	9610	4.3	1 visit	118 815	52.9
45-54	17 169	7.6	2 visits	60 473	26.9
55-64	42 689	19.0	3-4 visits	34 559	15.4
65-74	96 225	42.9	5 or more visits	10 738	4.8
75-84	40 667	18.1	Provider history		
85 plus	18 225	8.1	Less than 1 year	69 246	30.8
Female	13 399	6.0	1-3 years	63 223	28.2
Education			3-5 years	40 897	18.2
Did not finish high school	19 455	8.7	More than 5 years	51 219	22.8
High school graduate	69 452	30.9	Saw usual primary care provider	202 137	90
Some college	86 593	38.6			
College graduate or more	49 085	21.9			
Race					
White	182 350	81.2			
Black or African American	23 774	10.6			
Other	10 491	4.7			
Not reported	7970	3.6			
Hispanic or Latino	12 805	5.7			
Language other than English spoken at home	8784	3.9			
Elixhauser comorbid conditions					
Zero	78 179	34.8			
One	69 451	30.9			
Two	46 308	20.6			
Three or more	30 647	13.7			
General health: very good/excellent	62 328	27.8			
Mental health: very good/excellent	106 816	47.6			

^a^ n = 224 585.

**Table 2.** Distribution of Location Characteristics From VA Databases.^a^

	M	SD
Patient factor		
Patients residence rural	51%	34%
Provider factor		
Continuity primary care provider	81%	10%
Practice factors		
Wait times (days)		
New patients primary care	20.6	10.5
Established patients primary care	6.3	5.8
New patients mental health	13.2	7.0
Established patients mental health	3.9	3.8
New patients specialty care	17.8	10.6
Established patients specialty care	6.5	6.2
Third next available primary care (days)	7.51	7.61
Same day appointments (%)	57%	21%
Postdischarge contact within 2 days (% of discharges)	65%	21%
Nontraditional encounter (% of encounters)	21%	9%
# Emergency dept visit/primary care patient	.22	.24
Support staff ratio (per provider full-time equivalent)	3.31	.87
Panel size observed/expected	.85	.22
	N	%
Community-based outpatient clinic	721	84
Complexity: Most	565	66
Complexity: Moderate	138	16
Complexity: Least	151	18

Abbreviations: dept, department; SD, standard deviation, VA, Veterans Health Administration.

^a^ n = 854.

Overall, 51.6% of patients responded “always” for urgent care and 60.7% for routine care. Table 3 displays the adjusted ORs and CIs for effects of the independent variables on the 2 questions on timeliness. Sensitivity tests examining quartile values of location level measures are reported in Supplemental Appendix A, which confirmed the associations seen in Table 3. We observed a similar pattern of findings for both measures. The strongest association for patient factors was age, with higher ratings as age increased. Patients who reported having “very good” or “excellent” general health and mental health also provided more favorable ratings. A positive relation was found based for patients with 1 or more health comorbidities with ORs increasing stepwise from 1.03 to 1.07 for urgent care and 1.11 to 1.15 for routine care. Smaller associations were seen for education and race while no significant associations were observed for gender, ethnicity, or language spoken at home.

**Table 3.** Multilevel Logistic Results for Survey Questions on Whether Patients Got Primary Care Appointments as soon as Needed.^a^

Measure	Urgent care Always = 51.6%		Routine care Always = 60.7%	
	OR	95% CI	OR	95% CI
Patient factors				
Age: Less than 45	**.73**	.68-.78	**.76**	.72-.81
Age: 45-55	1.00		1.00	
Age 55-64	**1.21**	1.15-1.28	**1.21**	1.16-1.26
Age: 65-74	**1.39**	1.33-1.46	**1.39**	1.33-1.44
Age: 75-84	**1.57**	1.48-1.66	**1.56**	1.50-1.60
Age: 85 plus	**1.73**	1.61-1.86	**1.75**	1.67-1.84
Female	.99	.94-1.05	1.03	.99-1.07
Race: African American	1.00		1.00	
Race: White	.96	.92-1.01	**1.03**	1.00-1.07
Race: Other	**.90**	.84-.96	**.88**	.84-.93
Race: Not reported	**.85**	.79-.92	**.87**	.82-.93
Hispanic	1.05	.99-1.11	1.00	.96-1.05
Language other than English spoken at home	.99	.92-1.06	**.92**	.87-.97
Education: high school graduate	1.00		1.00	
Education: some college	**.92**	.89-.95	**.94**	.92-.96
Education: college graduate or more	.99	.96-1.03	1.01	.98-1.04
Education: non-graduate/unknown	1.02	.97-1.07	1.01	.97-1.05
General health (very good/excellent)	**1.75**	1.68-1.81	**1.61**	1.57-1.65
Mental health (very good/excellent)	**1.46**	1.42-1.51	**1.50**	1.47-1.54
Elixhauser group: 1 condition	**1.03**	1.00-1.06	**1.11**	1.09-1.14
Elixhauser group: 2 conditions	**1.04**	1.00-1.08	**1.12**	1.09-1.15
Elixhauser group: 3 or more conditions	**1.07**	1.03-1.12	**1.15**	1.11-1.18
Percentage patients with rural residence	**1.04**	1.01-1.07	**1.08**	1.06-1.11
Provider factors				
Provider visits: 1	**.84**	.81-.86	**1.15**	1.12-1.17
Provider visits 2	1.00		1.00	
Provider visits: 3 to 4	1.03	.99-1.06	**.95**	.92-.98
Provider visits: 5 or more	**1.21**	1.15-1.27	**1.06**	1.02-1.11
Provider history: Less than 1 year	**1.11**	1.07-1.15	**1.05**	1.03-1.08
Provider history: 1-2 years	1.00			
Provider history: 3-5 years	**1.08**	1.04-1.13	**1.06**	1.03-1.09
Provider history: 5 plus years	**1.28**	1.23-1.33	**1.23**	1.20-1.26
Saw usual primary care provider	**1.61**	1.53-1.70	**1.44**	1.40-1.49
Practice factors				
Primary care new patient wait	**.88**	.86-.90	**.90**	.88-.92
Primary care established patient wait	**.96**	.94-.99	**.95**	.93-.97
Mental health new patient wait	**.96**	.93-.98	.98	.96-1.00
Mental health established patient wait	1.01	.99-1.04	1.00	.97-1.02
Specialty care new patient wait	1.00	.98-1.03	.99	.97-1.01
Specialty care established wait	1.01	.98-1.03	1.00	.98-1.02
Third next available	.99	.97-1.02	1.01	.99-1.03
Same day appointments	**1.05**	1.03-1.08	**1.03**	1.00-1.05
Community-based outpatient clinic	.98	.80-1.05	1.01	.95-1.08
Continuity primary care provider	**1.06**	1.04-1.09	**1.07**	1.05-1.09
Post discharge contact within 2 days	**1.04**	1.01-1.07	**1.04**	1.02-1.07
Emergency department use	**1.04**	1.01-1.07	**1.03**	1.00-1.06
Non-traditional encounters	1.02	.99-1.04	1.01	.99-1.04
Support staff ratio	**1.02**	1.00-1.04	1.01	.99-1.03
Panel size (observed/expected)	**.95**	.93-.97	**.97**	.95-.99
Complexity group: Moderate	1.03	.95-1.11	.99	.92-1.06
Complexity group: Highest	1.04	.97-1.11	1.03	.97-1.09
VISN 1	1.00		1.00	
VISN 2	**.80**	.70-.91	**.80**	.72-.89
VISN 4	.89	.77-1.02	.93	.83-1.04
VISN 5	**.69**	.59-.81	**.67**	.59-.76
VISN 6	**.60**	.52-.70	**.57**	.50-.65
VISN 7	**.57**	.50-.66	**.59**	.52-.67
VISN 8	**.59**	.52-.68	**.62**	.56-.70
VISN 9	**.65**	.56-.76	**.65**	.58-.74
VISN 10	**.63**	.55-.71	**.65**	.59-.72
VISN 12	**.69**	.60-.80	**.75**	.67-.85
VISN 15	**.68**	.59-.79	**.79**	.70-.89
VISN 16	**.61**	.53-.70	**.59**	.53-.66
VISN 17	**.55**	.47-.63	**.56**	.50-.63
VISN 18	**.48**	.41-.56	**.47**	.42-.54
VISN 19	**.57**	.50-.65	**.56**	.50-.62
VISN 20	**.52**	.45-.61	**.54**	.48-.62
VISN 21	**.62**	.54-.71	**.61**	.55-.69
VISN 22	**.59**	.50-.69	**.61**	.53-.70
VISN 23	**.88**	.76-1.00	**.82**	.73-.91

Abbreviations: OR, odds ratio; VISN, veterans integrated service network.

^a^ Boldface values indicate *P* < .05.

Regarding provider factors, patients who saw their regular provider were more likely to provide favorable ratings on both urgent care (OR = 1.61) and routine care (OR = 1.44). Odds ratios increased as the duration of the relationship with their primary care provider increased, with OR of 1.28 for urgent visits and 1.23 for routine visits when the relationship had lasted 5 or more years. Odds ratio also increased as the number of visits with their usual provider increased. Measures of practice-level continuity with assigned primary care provider also were associated with higher ratings. A similar finding emerged for postdischarge contact within 2 days (OR = 1.04).

Patients in locations with a longer wait times for established primary care patients were less likely to provide favorable ratings for urgent care (OR = .96) and routine care (OR = .95). A similar pattern was observed when considering new patient wait times for care needed right away (OR = .88) and routine care (OR = .90). Although there was a small association of longer wait times with lower ratings for new mental health patients seeking care for urgent problems, there were no significant associations with mental health or specialty care wait times. Locations with a higher number of same day appointments were associated with more favorable patient ratings on access for urgent care (OR = 1.05) and routine care (OR = 1.03).

Regarding other practice-level factors, locations with more support staff per provider saw slightly more favorable urgent care ratings (OR = 1.02). Locations with a higher panel size per provider were associated with both lower urgent care (OR = .95) and routine care ratings (OR = .97). Compared to the New England regional network, patients seen in other regional networks were less likely to report favorable ratings on access. We tested region as a fixed effect finding an unconditional means model indicated region accounted for 1.6% of the variance in access, while division accounted for between 3.8% and 4.1%.

## Discussion

Once again, this analysis reveals that patient ratings of their health care experiences are a complex phenomenon. Ratings represent the degree of correspondence between patient preferences and expectations with perceived experience. The results showed strong influence of some, but not all, demographic variables on patient ratings. Findings point to the critical importance of continuing to use risk adjustment for demographic factors in research and when comparing results across different organizations and when using for accountability or pay for performance. It is also of interest that no differences were observed between male or female patients for this area, contrary to another study showing female patients VA providing lower ratings on communication, trust, and care quality (18). Increasing age had a strong effect on higher ratings of access, a finding noted in many studies of patient experience, including primary care (19–21). Some research has shown that patient expectations and preference change with age. For example, younger patients had a preference for shorter wait times, while older patients were more accepting of longer wait times for orthopedic procedures (22). It was speculated that this may be due to greater daily activity among younger patients leading to more interest in being seen earlier. Furthermore, as patients age, they may have more chronic health conditions, for which rapid medical attention may be less important. Health care systems could use approaches such as focus groups to better understand preferences in different age-groups and tailor interventions to best address their different needs.

As expected, we found a correlation, albeit moderate, between primary care wait times and ratings of the timeliness of appointments. We hypothesized that patients might also be influenced by their overall experience in their health care system and provide higher ratings if they experienced short waits for mental health and specialty care appointments within the same system of care. However, these, for the most part, did not influence the patients’ ratings for primary care timeliness. Nevertheless, the findings do reaffirm the importance of improving wait times within primary care for both urgent and routine care, an issue which has received considerable attention and for which practical guides are available (23).

Despite the identification of timeliness as a key dimension of health care quality, few health care systems measure and report actual appointment wait times. With the widespread adoption of electronic medical record systems with integrated scheduling, such measurement should be possible. This could provide support for improvement efforts in outpatient timeliness of care and offer another step an organization could take in efforts to improve patient experience.

A key finding is the effect of continuity with their primary care provider. Several measures of continuity were strongly associated with higher patient ratings of timeliness, independent of actual wait times. Seeing one’s primary care provider had the strongest association with positive ratings. Its effect is greater than that of the measures of appointment timeliness. Duration of relationship greater than 1 to 2 years also had a strong, stepwise association, although patients seeing new providers also rated their experience more highly. This may be related to the emphasis placed in the VA on ensuring new patients are seen quickly.

Practice-level measure of primary care continuity was also a positive factor. Finally, site level measures of frequency of postdischarge contact within 2 days by the primary care team were also significant. Such contact may be perceived by patients as contributing to a sense of continuity.

We interpret the moderate and consistent association to suggest that patients seen in VA clinics may be willing to trade-off longer wait times in order to see their primary care provider. Continuity is one of the core characteristics of high-quality primary care (24,25). Studies have shown higher levels of continuity are related to a better patient–provider relationship, greater overall patient satisfaction, improved uptake of preventive care, enhanced adherence to treatment, improved survival more accessible health care, and reduced health care use and costs (26–34). Thus, efforts at improving continuity could impact not only patient ratings of appointment timeliness but have positive effects across other domains. Conversely, it is also important that efforts to improve timeliness focusing on reducing appointment wait times avoid negative impact on continuity. Some current recommendations on improving access, such as setting up walk-in services or using midlevel providers to provide coverage, may be effective in reducing waits for some conditions but result in decreased continuity (35).

With regard to other practice factors, there was a small but statistically significant effect of the use of nontraditional encounters, including virtual hubs, secure messaging, telehealth visits, or group visits. All these interventions were designed in part to reduce demand for traditional face to face appointments. As technologies expand how patients can access health care, focused research on how these new technologies affect perceptions of access will be important (36).

We also found that smaller panel sizes and greater level of support staff were related to positive access perceptions. This suggests the need to balance supply and demand as a strategy to improve access. Finally, there were impressive differences across the different regional networks, even after controlling for the multiple variables discussed. This suggests there are many variables beyond the ones we were able to study which may be at play. The overall attitude of the patients to their health care system, including trust and satisfaction, may be among them.

A limitation of this analysis is that data were drawn exclusively from VA. The relationship between veterans and the VA health care system and expectations among its patients may differ from other systems. It would be worthwhile to replicate this analysis in other settings. The analysis also focused on 2 widely used survey questions. However, other surveys ask about access with differently worded questions, which might yield different results (37). Another limitation is the fact that practice-level data were used for several important variables including wait times. Additional or stronger associations may have been identified if we had been able to capture data for all independent variables at the level of the individual patient and provider. Finally, survey data may be affected by nonresponse bias, although our large sample size may ameliorate this concern.

In summary, in addition to multiple demographic variables and primary care appointment wait times, continuity with primary care provider was strongly associated with higher patient ratings of timeliness of primary care appointments. This suggests improvement efforts could focus on both wait times and continuity, and that efforts at improving wait times should be careful not to negatively impact continuity. Measurement and reporting of appointment wait times are uncommon in the United States but could also contribute to understanding and improving patient perceptions of appointment timeliness.

## Supplemental Material

Supplemental Material, Appendix_A - Factors Associated With Patient Ratings of Timeliness of Primary Care Appointments Click here for additional data file. Supplemental Material, Appendix_A for Factors Associated With Patient Ratings of Timeliness of Primary Care Appointments by Michael Mayo-Smith, Laurel E. Radwin, Hassen Abdulkerim and David C. Mohr in Journal of Patient Experience
