# Supplementary material for: Factors Associated With Patient Ratings of Timeliness of Primary Care Appointments
Source: J Patient Exp. 2020 Oct 27;7(6):1203–10. doi: 10.1177/2374373520968979 (PMC7786731; doi:10.1177/2374373520968979)
Supplement: Supplemental Material, Appendix_A - Factors Associated With Patient Ratings of Timeliness of Primary Care Appointments [file Appendix_A.pdf]

Appendix A

Multilevel logistic results for survey questions on whether patient got primary care appointments as soon as needed.

	Urgent care (N=98,484) Always=51.6%		Routine care (N= 203,884) Always= 60.7%	
Measure	OR	95% CI	OR	95% CI
**Patient factors**				
Age: Less than 45	**.73**	.68 - .79	**.77**	.72 - .81
Age: 45 to 55	1.00		1.00	
Age 55 to 64	**1.21**	1.15 - 1.28	**1.21**	1.16 - 1.26
Age: 65 to 74	**1.39**	1.33 - 1.46	**1.39**	1.34 - 1.44
Age: 75 to 84	**1.57**	1.48 - 1.66	**1.57**	1.50 - 1.63
Age: 85 plus	**1.73**	1.61 - 1.86	**1.76**	1.67 - 1.84
Female	.99	.94 - 1.05	1.03	.99 - 1.07
Race: African American	1.00		1.00	
Race: White	**.90**	.84 - .96	**.88**	.84 - .93
Race: Other	**.85**	.79 - .92	**.87**	.82 - .93
Race: Not reported	.97	.93 - 1.01	**1.03**	1.00 - 1.07
Hispanic	1.05	.99 - 1.11	1.00	.96 - 1.05
Language other than English spoken at home	.99	.92 - 1.06	**.92**	.87 - .97
Education: high school graduate	1.00		1.00	
Education: some college	.92	.89 - .95	.94	.92 - .96
Education: college graduate or more	.99	.96 - 1.03	1.01	.98 - 1.04
Education: non-graduate/unknown	1.02	.97 - 1.07	1.01	.97 - 1.05
General health (very good/excellent)	**1.75**	1.69 - 1.81	**1.61**	1.57 - 1.65
Mental health (very good/excellent)	**1.46**	1.42 - 1.51	**1.50**	1.47 - 1.54
Elixhauser group: one condition	**1.03**	1.00 - 1.06	**1.11**	1.08 - 1.14
Elixhauser group: two conditions	**1.04**	1.00 - 1.08	**1.12**	1.09 - 1.15
Elixhauser group: three or more conditions	**1.07**	1.03 - 1.12	**1.15**	1.11 - 1.18
Percent patients with rural residence – Q2	1.06	.99 - 1.14	**1.08**	1.02 - 1.14
Percent patients with rural residence – Q3	**1.08**	1.00 - 1.16	**1.13**	1.06 - 1.20
Percent patients with rural residence – Q4	**1.14**	1.05 - 1.24	**1.24**	1.16 - 1.33
**Provider factors**				
Provider visits:1	**.84**	.81 - .86	**1.15**	1.12 - 1.17
Provider visits 2	1.00		1.00	
Provider visits: 3 to 4	1.03	.99 - 1.06	**.95**	.92 - .98
Provider visits: 5 or more	**1.21**	1.15 - 1.27	**1.07**	1.02 - 1.12
Provider history: Less than 1 year	**1.11**	1.08 - 1.15	**1.06**	1.03 - 1.08
Provider history: 1-2 years	1.00		1.00	
Provider history: 3 to 5 years	**1.08**	1.04 - 1.13	**1.06**	1.03 - 1.09
Provider history: 5 plus years	**1.28**	1.23 - 1.33	**1.23**	1.20 - 1.26
Saw usual primary care provider	**1.61**	1.53 - 1.70	**1.44**	1.40 - 1.49

Appendix A continued

	Urgent care (N=98,484) Always=51.6%		Routine care (N= 203,884) Always= 60.7%	
Measure	OR	95% CI	OR	95% CI
Primary care new patient wait Q2	**.91**	.85 - .98	**.92**	.87 - .97
Primary care new patient wait Q3	**.86**	.80 - .92	**.87**	.82 - .92
Primary care new patient wait Q4	**.72**	.67 - .78	**.75**	.70 - .79
Primary care established patient wait Q2	.99	.92 - 1.06	.99	.93 - 1.05
Primary care established patient wait Q3	.94	.87 - 1.01	**.91**	.85 - .97
Primary care established patient wait Q4	**.89**	.82 - .97	**.85**	.79 - .91
Mental health new patient wait Q2	1.00	.94 - 1.07	.98	.93 - 1.03
Mental health new patient wait Q3	.97	.91 - 1.03	.98	.93 - 1.04
Mental health new patient wait Q4	.92	.86 - 0.99	.96	.91 - 1.02
Mental health established patient wait Q2	1.02	.95 - 1.09	.99	.94 - 1.05
Mental health established patient wait Q3	1.01	.95 - 1.08	.97	.92 - 1.03
Mental health established patient wait Q4	1.03	.95 - 1.11	.99	.94 - 1.06
Specialty care new patient wait Q2	1.03	.96 - 1.11	1.01	.95 - 1.07
Specialty care new patient wait Q3	.98	.91 - 1.05	.95	.89 - 1.01
Specialty care new patient wait Q4	.99	.92 - 1.07	.96	.91 - 1.02
Specialty care established patient wait Q2	.96	.89 - 1.03	.94	.89 – 1.00
Specialty care established patient wait Q3	.99	.92 - 1.07	.97	.91 - 1.03
Specialty care established patient wait Q4	1.03	.95 - 1.11	1.00	.94 - 1.07
Third next available Q2	.97	.91 - 1.04	.97	.92 - 1.03
Third next available Q3	1.01	.94 - 1.08	1.00	.94 - 1.06
Third next available Q4	.99	.92 - 1.07	1.02	.96 - 1.09
Same day appointments Q2	1.00	.94 - 1.07	1.02	.96 - 1.07
Same day appointments Q3	**1.10**	1.03 - 1.18	**1.06**	1.00 - 1.13
Same day appointments Q4	**1.13**	1.05 - 1.23	1.06	.99 - 1.13
Community-based outpatient clinic	.99	.91 - 1.07	1.01	.94 - 1.08
Continuity primary care provider Q2	1.07	.99 - 1.15	**1.07**	1.01 - 1.14
Continuity primary care provider Q3	**1.12**	1.03 - 1.22	**1.16**	1.08 - 1.24
Continuity primary care provider Q4	**1.21**	1.11 - 1.32	**1.22**	1.14 - 1.31
Post discharge contact within 2 days Q2	**1.07**	1.00 - 1.15	**1.08**	1.02 - 1.14
Post discharge contact within 2 days Q3	1.04	.97 - 1.11	**1.06**	1.00 - 1.13
Post discharge contact within 2 days Q4	**1.10**	1.02 - 1.18	**1.10**	1.04 - 1.17
Emergency department use Q2	.94	.87 - 1.03	.98	.92 - 1.05
Emergency department use Q3	1.01	.92 - 1.10	1.01	.94 - 1.09
Emergency department use Q4	**1.09**	.99 - 1.20	**1.12**	1.03 - 1.21
Non-traditional encounters Q2	1.01	.94 - 1.08	1.02	.96 - 1.07
Non-traditional encounters Q3	1.02	.95 - 1.09	1.02	.96 - 1.08
Non-traditional encounters Q4	**1.07**	1.00 - 1.16	**1.08**	1.02 - 1.15

Appendix A Continued

	Urgent care (N=98,484) Always=51.6%		Routine care (N= 203,884) Always= 60.7%	
Measure	OR	95% CI	OR	95% CI
Support staff ratio Q2	1.05	.98 - 1.12	1.01	.96 - 1.07
Support staff ratio Q3	1.02	.95 - 1.09	.98	.93 - 1.04
Support staff ratio Q4	1.05	.98 - 1.12	1.03	.97 - 1.08
Panel size (observed/expected) Q2	.99	.93 - 1.05	1.01	.95 - 1.06
Panel size (observed/expected) Q3	.95	.89 - 1.02	.96	.91 - 1.01
Panel size (observed/expected) Q4	.93	.86 – 1.00	.95	.90 - 1.01
Complexity group: Moderate	1.00	.89 - 1.11	.94	.86 - 1.03
Complexity group: Highest	1.02	.92 - 1.13	.99	.91 - 1.08
VISN 1	**1.00**		**1.00**	
VISN 2	**.80**	.69 - .92	**.79**	.71 - .89
VISN 4	**.84**	.72 - .98	**.88**	.78 - .99
VISN 5	**.67**	.57 - .79	**.63**	.55 - .72
VISN 6	**.57**	.48 - .67	**.54**	.47 - .62
VISN 7	**.56**	.48 - .65	**.57**	.50 - .65
VISN 8	**.58**	.50 - .67	**.60**	.54 - .68
VISN 9	**.61**	.52 - .72	**.60**	.53 - .68
VISN 10	**.61**	.53 - .71	**.63**	.56 - .71
VISN 12	**.67**	.57 - .78	**.72**	.63 - .81
VISN 15	**.64**	.55 - .75	**.72**	.64 - .82
VISN 16	**.61**	.52 - .70	**.59**	.52 - .66
VISN 17	**.53**	.46 - .62	**.54**	.47 - .61
VISN 18	**.49**	.41 - .57	**.47**	.41 - .54
VISN 19	**.55**	.48 - .64	**.55**	.49 - .62
VISN 20	**.50**	.42 - .59	**.52**	.45 - .60
VISN 21	**.62**	.54 - .71	**.59**	.53 - .67
VISN 22	**.54**	.45 - .64	**.54**	.47 - .62
VISN 23	**.86**	.74 – 1.00	**.79**	.70 - .89

Note: Bold font indicates $p<.05$. Q represents “quartile” where Q4 is upper quartile
